# European radiographers’ challenges from mammography education and clinical practice – an integrative review

**DOI:** 10.1007/s13244-016-0542-1

**Published:** 2017-03-16

**Authors:** Eija Metsälä, Nicole Richli Meystre, José Pires Jorge, Anja Henner, Tiina Kukkes, Cláudia Sá dos Reis

**Affiliations:** 1 Metropolia University of Applied Sciences, Mannerheimintie 172, PO BOX 4033, 00079 Metropolia, Finland; 2 Haute Ecole de Santé Vaud, Av. de Beaumont 21, 1011 Lausanne, Switzerland; 3 Oulu University of Applied Sciences, Kiviharjuntie 8, 90220 Oulu, Finland; 4 Tartu Health Care College, Nooruse 5, 50411 Tartu, Estonia; 5 Escola Superior de Tecnologia da Saúde de Lisboa, Av. D. João II, Lote 4.69.01, 1990-096 Lisboa, Portugal; 6 Department of Medical Radiation Science, Curtin University, GPO Box U1987, Perth, WA 6845 Australia

**Keywords:** Breast cancer, Mammography, Education, Clinical practice, Europe

## Abstract

**Objectives:**

This study aims to identify European radiographers’ challenges in clinical performance in mammography and the main areas of mammography that require more and better training.

**Methods:**

An extensive search was performed to identify relevant studies focused on clinical practice, education and training in mammography published between January 2010 and December 2015 in the English language. The data were analysed by using deductive thematic analysis.

**Results:**

A total of 27 full text articles were read, evaluating their quality. Sixteen articles out of 27 were finally selected for this integrative review. The main challenges of radiographers’ mammography education/training can be divided into three groups: training needs, challenges related to radiographers, and challenges related to the organization of education. The most common challenges of clinical performance in mammography among European radiographers involved technical performance, the quality of practices, and patient-centeredness.

**Conclusions:**

The introduction of harmonized mammography guidelines across Europe may serve as an evidence-based tool to be implemented in practice and education. However, the variability in human and material resources as well as the different cultural contexts should be considered during this process.

***Teaching Points*:**

*• Radiographers’ awareness of their professional identity and enhancing multiprofessional cooperation in mammography.*

*• Radiographers’ responsibilities regarding image quality (IQ) and optimal breast imaging performance.*

*• Patient-centred mammography services focusing on the psychosocial needs of the patient.*

*• Challenges: positioning, QC-testing, IQ-assessment, optimization of breast compression, communication, teamwork, and patient-centred care.*

*• Introduction of evidence-based guidelines in Europe to harmonize mammography practice and education.*

## Introduction

Mammography remains the imaging modality that has proved its cost-effectiveness for screening and diagnosis of breast diseases [1, 2]. For that reason, it is one of the X-ray examinations most frequently performed for the healthy female population in the world. Due to the radiation, mammography requires optimized practice [3]. In order to ensure this, quality standards must be implemented and followed. These standards should address all aspects in the mammography examination, including equipment, performance, provider profile, and healthcare staff. Monitoring the quality standards provides opportunities to improve practice through a feedback mechanism that allows the identification of sub-optimal practice, and can suggest difficulties, training needs, and challenges [4–10].

Radiographers face challenges in clinical practice due to upgrades in technology, equipment variability, the introduction of new techniques, the culture of each healthcare institution, patient interaction, and the requirements for continuous professional development [11, 12]. To follow all updates and to be aware of all requirements, education and training are crucial, although the attendance can be affected by costs, work constraints, geographical location, timing, quality issues, family constraints, a perceived lack of benefits, and a lack of relevance, support and availability of education and training regarding the several needs [11].

The quality of breast cancer screening can be defined by using several quality outcomes. The European Guidelines for quality assurance in breast cancer screening and diagnosis mention 39 key performance indicators for mammography screening [13]. The quality of breast cancer screening can roughly be divided into the (screening) process quality, the quality of practices, and technical quality. *The quality of the screening process* comprises the invitation to the screening programme, the justification and optimization of mammography examinations, the staff training and qualification requirements, recording and reporting examination data, as well as the attendance rates [14, 15]. Patients’ wellbeing and health is the ultimate goal of any screening programme. *Patient-centeredness* and especially patient-provider communication is known to be associated with attendance rates of screening programmes [16]. This aspect is also central in the European Guidelines for quality assurance in breast cancer screening and diagnosis [13]. *The quality of practices* comprises the determination of patient doses and their comparison with the diagnostic reference levels, clinical image quality assessment, self-assessment, and clinical audits. *Technical quality assurance* consists of acceptance testing and quality control during the use of equipment. This quality dimension considers image acquisition, image processing, and imaging display [14, 15]. Due to the emphasis on patient-centeredness as an important component of screening process quality, it was chosen as the framework for deductive thematic analysis in order to widely cover the aspects of breast cancer screening quality. In this integrative review, the quality of breast imaging service was also considered from the viewpoints of staff education (radiographers), technical quality, and the quality of practices.

The aim of this study was to identify European radiographers’ challenges in clinical performance in mammography and the main areas of mammography that require more and/or improved training.

The following search questions were set:

1. What are the most common challenges of mammography training for radiographers?
2. What are the biggest challenges for radiographers from the viewpoints of (1) technical performance, (2) quality of practices, and (3) patient-centeredness in the breast imaging service?

## Materials and methods

An extensive search was performed to identify relevant studies focused on clinical practice as well as on education and training in mammography published in the English language between January 2010 and December 2015. The Population Intervention Context Outcomes (PICO) strategy (Table 1) was used for the construction of research questions and for the bibliographical search. It is reported to facilitate the definition of population groups, interventions, comparators, and outcomes of interest. It was also used as support to define the inclusion and exclusion criteria. For the comparison (C), it is possible to replace it with the identification of context (Co) which is relevant, especially in (non-comparative) qualitative studies [17].

**Table 1:** The PICOs in search questions 1 and 2

The PICO for question 1	The PICO for question 2
***P*** *opulation*: radiographer	***P*** *opulation*: radiographer
***I*** *ntervention:* mammography education	***I*** *ntervention:* breast imaging service
***C*** *ontext:*	***C*** *ontext:*
***O*** *utcomes of Interest:* challenges	***O*** *utcomes of Interest:* challenges of technical performance quality of practices patient-centred services

### Evidence review strategy

The integrative review method that allows combining studies with a variety of research designs was used [18]. The following electronic search engines and databases were used: EBSCO Host: Academic Search Elite; CINAHL with Full Text; CINHAL, and Science Direct. We also searched Pro Quest: ABI/INFORM Complete; Applied Social Sciences Index and Abstracts (ASSIA); Biological Sciences; British Humanities Index (BHI); ERIC and MEDLINE and the Open Access Theses and Dissertations database (OATD). According to authors’ previous experience in database search in the field, the most relevant findings can be found by using keywords and their combinations with the command that these must either be found in the title or abstract of the article. The subject headings and keywords searched were teaching OR learning OR education; radiographer (with related/allied search option) OR radiologic technologist; mammography OR breast screening; challenge; quality OR image quality; patient-centred; and evaluation. These subject headings and keywords were used in similar combinations in the several databases, and with the option that these must either be found in the title or abstract of the article if that was possible in the particular database.

*Inclusion criteria* for the selected studies were the focus on European radiographers’ work in breast screening or clinical mammography, population based or opportunistic screening, describing the challenges of the breast imaging service or education, technical aspects, quality of practice, and the patient-centred viewpoints on mammography. We included qualitative and quantitative peer-reviewed studies, intervention studies, and research and development projects with sound methodology comprising the JBI levels of evidence for effectiveness from 1 to 3 and the levels of evidence for meaningfulness from 1 to 3 [19]. The search was limited to papers published in 2010 or later since the studies published before that year may not be relevant as the imaging technology and quality assurance practices are developing fast.

The search was performed in the databases between 1 October and 1 November 2015, looking through all the titles and selecting the relevant titles for the abstract search. Two reviewers then studied the abstracts and the full texts independently. The relevant articles were chosen within the full text review based on a consensus discussion. The agreement percentage was 81% (22/27) before the consensus discussion and 100% after the discussion. The reviewers evaluated the quality of the reporting in the studies independently according to the modified version of the STROBE v4 checklist for cohort, case–control and cross-sectional studies (combined) [20]. This type of evaluation criteria has previously been used in several published integrative reviews [21–23]. Detailed evaluation of the quality of each study’s methodology and conduct was not performed because the focus of the review was rather to identify the dimensions than the effect of the phenomenon. It aimed to identify challenges in training and practice in the field. The studies that did not achieve the JBI levels of evidence for effectiveness 1–3 and the levels of evidence for meaningfulness 1–3 were excluded. Also, the studies receiving two or more ‘hardly or not at all satisfies assessment criteria’ scores in the STROBE checklists were rejected. However, these evaluation methods were consistent, i.e., a study that got two or more ‘hardly or not at all satisfies assessment criteria’ scores, rarely reached the JBI level 3. In case of a mismatch in quality evaluations, a consensus was discussed (Table 2).

**Table 2:** Critical assessment of the reporting of the studies

Ref.	Assessment criteria of the studies.									
	1.	2.	3.	4.	5.	6.	7.	8.	9.	10.
[5]	**	*	**	**	*	*	**	**	_	**
[33]	*	**	**	**	*	*	*	*	**	**
[42]	**	*	**	**	**	*	*	**	x	**
[34]	*	*	*	**	*	**	**	**	*	**
[32]	**	*	**	**	*	*	*	**	*	**
[45]	**	**	**	*	*	**	*	**	**	**
[36]	**	*	**	*	**	**	*	**	*	*
[35]	**	*	**	*	**	*	_	**	*	**
[40]	**	**	**	**	**	**	*	**	*	**
[41]	**	*	**	**	**	**	**	**	*	**
[37]	**	*	**	**	*	**	*	**	**	**
[38]	**	*	**	**	*	**	**	*	*	**
[39]	**	*	**	*	*	*	*	**	*	**
[43]	**	**	**	**	*	**	**	**	*	**
[30]	**	**	**	**	x	**	x	**	*	*
[44]	**	*	**	*	*	**	*	**	_	*

1. Study background and theoretical framework are clearly defined.

2. Purpose, aim and research questions are clearly defined.

3. The design is clearly stated.

4. The setting is clearly described.

5. For independent and dependent variables, confounders are clearly identified and consistently implemented or something else should be added here.

6. Data sources and analysis methods are clearly described.

7. Efforts to address potential sources of bias are described.

8. Research questions are answered logically.

9. Study limitations and generalizability are discussed.

10. Relevance to the topic.

** assessment criteria are satisfied

* assessment criteria are partly satisfied

_ assessment criteria are hardly or not at all satisfied

x assessment criteria do not apply.

The shortened version of the STROBE checklist was used due to the need to include studies with several types of designs into this integrative review. In addition, the use of this strategy allows commensurable evaluation of all the studies, showing the evaluation results in a table format. The results of the selected studies were analysed using deductive thematic analysis, a suitable option because there were studies with different kinds of designs. The challenges of European breast imaging practices were categorized under three dimensions of breast imaging quality: a) technical performance, b) quality of practices, and c) patient-centred viewpoints. The first two dimensions have been defined in the theoretical background of this study [13, 14]. The third dimension, also mentioned in our PICO components, was clearly separate, stemming from the studies forming the data of this integrative review.

## Results

**Fig. 1:**
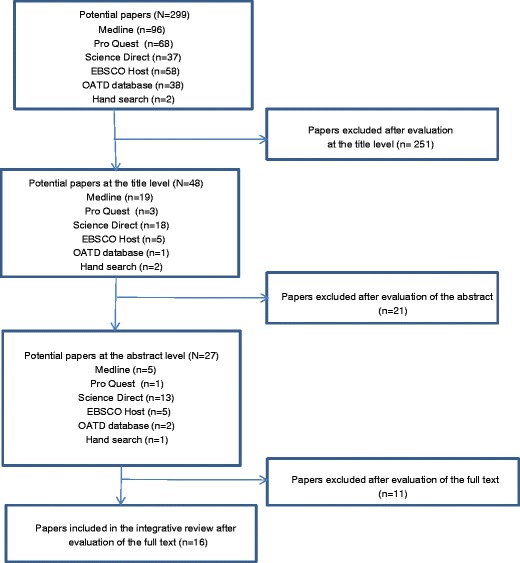
The selection process of the studies

With the selected keywords and their combinations, a total of 299 results were obtained using the following databases and search engines: Medline - 96, Pro Quest - 68, Science Direct - 37, EBSCO Host - 58, and OATD (theses and dissertations database) - 38. Two relevant titles were found by hand search. The most typical reason for exclusion was the lack of relevance for the search questions. Abstracts of 48 articles were read, and 21 were excluded after their evaluation. Reasons for exclusion at the abstract level were associated with the issues of relevance, the type of the article or the target group. A total of 27 full text articles were read to evaluate their quality. Sixteen articles out of 27 were finally selected for this integrative review (Fig. 1). Reasons for rejection at the evaluation of full text level involved a low reporting quality not satisfying the evaluation criteria mentioned in Table 2: one article was rejected based on criteria 1 and 8, two based on criteria 7 and 10, and also due to the focus on the technical performance of devices only or patient satisfaction only. One article was rejected based on criteria 3 to 6 and also because the target group was not radiographers (*n* = 1). One article, a case–control study not performed in Europe, was rejected because of JBI levels.

### Description of the selected studies

Seven out of the 16 selected studies had been performed in the United Kingdom, three in the Netherlands, two in Portugal, two in Serbia and/or Croatia, one in Turkey, one in Norway, and one involving several countries in Eastern Europe. Most of the studies were quantitative (*n* = 12), three studies were qualitative, and one study used a mixed method approach. In five out of the 16 selected studies, the equipment in use was screen-film mammography (SFM) [24] or SFM combined with digital mammography (DM) [5, 25–27]. Four of these research settings using SFM systems were located in Eastern Europe [5, 25, 26] (Table 3).

**Table 3:** Description of studies

Ref.	Country	Objective of the study	Study design, sample and setting	Measures and analysis methods	Most common challenges of mammography training for radiographers	Challenges of education classified	What are the biggest challenges radiographers meet in breast imaging from the aspects of a) technical performance b) quality of practices and c) patient-centeredness ?	Biggest challenges in breast imaging practice classified
[5]	Croatia and Serbia	To investigate the need for and achievements of a comprehensive QA programme in Croatia and in Serbia from technical and radiological viewpoints.	Pre test-post test intervention study comparing image quality (IQ) in Serbia (*n* = 684 images) and Croatia (*n* = 400 images). Hospitals had either film-screen (FS) or digital (DI) systems.	IQ, patient dose and equipment performance. Intervention was corrective action based on IQ evaluations. Quantitative analysis.	The study emphasized a need for training in cooperation of all the operating mammography staff (medical physicist with radiologists and radiology technologists).	1) topics where education should be targeted: multiprofessional co-operation viewpoint 2) to improve IQ.	a) cleaning of working surfaces, screens, cassettes and processing units, change from manual to AEC, service adjustment to AEC, change of exposure parameters, introduction of daily sensitometry tests and replacement of image receptors b) incorrect positioning, contrast problems, artefacts.	a) 1, 2, 3, 4, 5 b) 1, 2, 3
[25]	Croatia	To evaluate mammographic (MG) image quality and to identify the most common. deficiencies.	*N* = 420 MG examinations from 84 MG units participating in the Croatian nationwide breast cancer screening program were reviewed over a 6-month period. Technical data was collected by questionnaires.	Four image quality categories were rated using the image evaluating system based on the American College of Radiology and the European Commission proposals. Quantitative analysis.	Lack of training: additional training for RTs is necessary to improve IQ and make examination more comfortable for women which could directly increase the response rate to the screening in future.	topics where education should be targeted: 2) education for RTs to improve IQ 3) patient viewpoint (to make the examination more comfortable to patients).	a) problems with viewing conditions b) problems in positioning, compression, labelling, documentation, contrast, image artefacts, different viewpoints of RTs and radiologists about IQ. There was variation in IQ by organization, equipment, staff education, working habits and motivation and by economic interests.	a) 7, 8 b) 1, 3, 8
[33]	UK	To evaluate the feasibility of training sufficient for radiographers to deliver an intervention to promote early presentation of breast cancer to all older women attending for their final routine mammogram.	Mixed methods study assessing the feasibility of training of 25 radiographers in one breast screening service at key time points during the training process.	Measures: competence and confidence of radiographers to deliver the intervention, radiographers’ perceptions and experiences both of training and delivering the intervention. Qualitative interview study.	Commitment, motivation, varying levels of staff basic education and clinical experience, informing about education, giving regular feedback about performance.	4)radiographer related factors: commitment, motivation, varying basic education 5) challenges related to organizing education: informing about education, giving feedback.	c) How to take into account the promotion of breast screening adherence in radiographer’s work.	c) 3
[26]	Turkey	To evaluate the mammography image quality in İstanbul and to survey the awareness of mammography quality.	Cross-sectional study. *N* = 55/50 mammography units were assessed in İstanbul. Hospitals had either film-screen (FS) or digital (DI) systems.	An American College of Radiology (ACR) accreditation phantom was used to assess image quality. 10-item questionnaire for mammographers concerning the type of mammography equipment and IQ in their unit. Quantitative analysis.	Radiographer’s lack of awareness and knowledge of mammography image quality.	2) topic where education should be targeted: education for RTs to improve IQ.	a) artefacts on the phantom because of the absence of routine cleaning and quality control following daily, monthly and yearly QA protocols, b) poor positioning, various problems in image processing, unrealistic IQ ratings by radiographers, low image quality in images taken by analogue system compared to images taken by CR and DR systems.	a) 1, 3, 10 b) 1, 4, 7
[24]	Africa, Asia and Eastern Europe	To study mammography practice from an optimisation point of view by assessing the impact of simple and immediately implementable corrective actions on image quality.	Prospective multinational study that included 54 (FS) mammography units and more than 21,000 images in 17 countries.	Images were evaluated using a three-level image quality scoring system. Following initial assessment, appropriate corrective actions were implemented and image quality was re-assessed in 24 units.	Radiographer’s lack of training.	2) topic where education should be targeted: education for RTs to improve IQ .	a) cleaning of screens, cassettes, processing units and working surfaces, damaged or scratched image receptors, or film-screen combinations that are not spectrally matched, exposure technique by using AEC, compression and tube voltage settings, viewing conditions b) issues related to film processing, inappropriate radiographic techniques.	a) 1, 2, 5, 6, 7, 8, 10
[36]	UK	To explore the experiences of women attending for diagnostic tests prior to and after diagnosis in order to inform practice.	A qualitative, exploratory and longitudinal study design. A convenience sample (*n* = 16) was recruited throughout the North-East of Scotland.	*N* = 25 semi-structured interviews with 7 participants taking part in a single interview, a further 7 and 1 participants taking part in 2 and 3 interviews. Interviews were analysed by thematic approach.	Inability of radiographers to discuss the outcomes of breast imaging with patients.	5) topic where education should be targeted: patient centeredness: psychosocial aspects, patient comfort, communication, counselling.	c) Patients experience anxiety when awaiting test results. It is necessary for imaging departments to review their result-giving policy. This should include a multi-professional, multi-departmental approach to provide a timely effective standardised and seamless result-giving service for all, which reflects the use of modern communication methods.	c) 1
[37]	UK	To study if there was variation in compression force by a practitioner over time.	Retrospective study in a regional breast screening service in the North of England.	*N* = 500/344 clients/1032 mammogram sets by 14 practitioners FS mammograms from 2004, 2007 and 2010 screening rounds. Measures: compression force, b MGD (mean glandular dose). Quantitative analysis.			a) Compression force and thereby breast thickness and MGD varied by screening rounds and practitioners. When the same practitioner performed breast imaging at different rounds there was less variation.	a) 8
[27]	UK	To evaluate image quality, compression and dose in Irish symptomatic breast units.	Mixed methods study in 16 mammographic units, having either film-screen (FS) or digital (DI) systems. *N* = 4071 patient images from 1010 patients.	Measures: dose, breast density, compression force, compression level, IQ. Quantitative, quantification of qualitative data.			a) Little lower compression force than in EU recommendations, b) MGDs with FS were higher than those with DI systems, overall they were higher in symptomatic breast units than in Irish Breast Screening service, DRLs are defined for standard sized patients which does not always apply to reality.	a) 8 b) 5
[31]	Portugal	To characterise the technology for digital mammography installed in Portugal.	Mixed methods design. The sample was radiographers (*n* = 118 + 59) and radiologists (*n* = 69).	Practice was analysed using qualitative and quantitative methods. IQ (clinical and technical), patient dose and phantoms dose, equipment performance were evaluated with qualitative and quantitative methods.	Education and training needs were identified. Radiographers acknowledged the need of dedicated education and training on digital mammography (DM), training in artifact recognition, quality control, intervention, optimisation, dosimetry and tomosynthesis. Most radiographers had received training in DM but there was variation in type, amount and the organizer of training.	topics where education should be targeted: 2) education to improve IQ: artefact recognition, quality control and dosimetry; 6) further examinations such as interventions and tomosynthesis 7) performing DM 8) pathology.	a) Implementation of the quality control program and performance; variation in exposure parameters, dose and compression force; use of cassettes and image plates that are damaged b)positioning, repeat/reject analysis should be implemented to monitor the most typical failures in practice c) high workload promotes less time to be with the patient.	a) 4, 5, 8, 10, b) 1, 6 c) 2
[32]	UK, Norway	To characterise practices of healthcare staff (radiographers and radiologists) in digital mammography.	Quantitative comparative study in UK and Norway. In each of the two centres, mammograms from 112 women aged 50–70 years were reviewed.	Four radiographers from Norway with a degree in radiography and four from the UK, holding a degree in Radiography and a Postgraduate Certificate in Mammography, were invited to participate in the study. Quantitative analysis.	Training needs for radiographers in using PGMI criteria in a uniform way.	2) topic where education should be targeted: education for RTs to improve IQ.	Use of PGMI varied between centres in both number and interpretation of the criteria employed.	b) 9
[29]	Netherlands and United States	To assess the Mean Glandular Dose (MGD) to the breast in digital mammography in conditions relevant to clinical practice.	Retrospective study. Mammographic compressions of 9188 women (the NL dataset) and 1851 women from a breast imaging centre in Pittsburgh, PA (the US dataset).	Available parameters of a set of 37,518 mammographic compressions in Denmark and 7171 compressions in USA. Quantitative analysis.			a) The forces and pressures applied in the NL dataset were significantly higher than in the US dataset. c) Factors contributing to this variation include the pain threshold of the woman, the radiographer’s sensitivity for pain expression, the uncertainty or inaccuracy in estimating the pressure on the breast, the radiographer’s opinion of what is a good compression, and local conventions.	a) 8 c) 4
[38]	Netherlands	To compare pain, projected breast area, radiation dose and image quality between flexible (FP) and rigid (RP) breast compression paddles	The study was conducted in a Dutch mammographic screening unit (288 women).	Measures: pain experience, projected breast area, radiation dose. Image quality was reviewed by 3 radiologists and 3 radiographers.			a) Pain experience showed no difference between flexible and rigid breast compression paddles. Flexible paddles do not depict clinically relevant retroglandular areas as well. Flexible paddles move breast tissue from image area at the chest wall side. Rigid paddles depict more breast tissue and show better contrast. Rigid breast compression paddles are recommended for standard mediolateral-oblique and craniocaudal views.	a) 8
[28]	UK	To characterise image quality (IQ) in mammography using technical and clinical (realistic) breast models.	Data were collected from three consecutive screening events in three breast screening sites. Data comprised 975 clients (2925 client visits, 11 700 MG images).	Measures: practitioner code, applied compression force (N), breast thickness (mm), BIRADS density category. Quantitative analysis.			a) Practitioners across three breast screening sites behave differently in the application of compression force. There was also internal variability inside screening sites in compression force and thickness.	a) 8
[34]	Portugal	To develop an e-learning course on breast imaging for radiographers and evaluate the course.	RCT target group radiographers (*n* = 120) and radiology students (*n* = 70).	Measures: efficacy, effectiveness and user satisfaction. Quantitative analysis.	E-learning group got better scores in knowledge and course satisfaction. E-learning may be some complementary solution to solve challenges in learning breast imaging.			
[30]	UK	To study compression behaviours of practitioners during screening mammography.	Qualitative research with 6 focus group interviews (*n* = 41 practitioners) in six breast screening centres in England.	Interview focused on experiences of, the influence on behaviour of practitioners applying compression force in mammography.			a and c) viewpoints of practitioners’ use of compression during screening MG: patient empowerment, white lies, time for interactions, uncertainty of own practice, culture using compression force, power/medical dominance, compression controls, digital technology, dose audit-safety net and a numerical scale of compression force.	a) 8 c) 4
[35]	Netherlands	To compare mammography performed with and without radiolucent positioning sheets.	*N* = 184/179 women screened in the Dutch breast screening programme, providing written informed consent to have one additional image taken with positioning sheets.	Measures: projected breast area, image quality, pain experience and radiation dose. Quantitative analysis.			a) With positioning sheets significantly more pectoral muscle, lateral and medial breast tissue was projected (CC-views) and more and deeper depicted pectoral muscle (MLO-views). In contrast, visibility of white and darker areas was better on images without positioning sheets, radiologists were there for the better ability to detect abnormalities (MLO-views). Women experienced more pain with positioning sheets (MLO-views only)	a) 9 b) 1 c) 4

The focus of six selected studies was on the compression force and its association with dose [27–31]. Four studies evaluated image quality in mammography at national levels [5, 25, 27, 31], two studies at local levels [26, 32], and one study about image quality evaluation was multinational [24]. Three studies considered radiographer training related to mammography [31, 33, 34]. There was also a study focusing on the evaluation of clinical image quality [32], especially on positioning [35], and one study focusing on patient viewpoints of mammography examination [36] (Table 3).

### Challenges in mammography education

All the studies addressed radiographer training (to a greater or lesser extent), but all of them identified the need for additional training in a wide range of issues or contents in order to achieve high quality in mammography services.

Thematic analysis of the selected study results produced three main challenges of mammography training for radiographers: (1) training needs, (2) radiographer related challenges, and (3) challenges related to the organization of education. *Training needs* comprised four subthemes, each of them representing a specific area of training needing to be developed in mammography education: (1) multiprofessional cooperation within the diagnostic process of breast cancer (BC) [5]; (1) image quality such as artefact recognition, quality control and dosimetry as well as implementing and maintaining quality control and quality assurance [5, 24, 25, 31, 32]; (3) competences in patient-centred work such as counselling patients in the issues related to mammography or taking into account the psychosocial needs and comfort of the patient [25], and (4) performance of breast imaging in an optimal way, including breast pathology, and performing basic digital mammography and further examinations, e.g., interventional procedures of the breast tissue and tomosynthesis [31]. *Radiographer related challenges* mentioned in the selected studies included a lack of commitment and motivation, and the effective delivery of the training, particularly in relation to role extension associated with the amount of time required for radiographers to learn and rehearse the script [33]. *Challenges related to the organization of education* involved information about the education and giving feedback to those who attend these trainings [33] (Table 3).

### Challenges of mammography practice

The second PICO question based on deductive thematic analysis, *Challenges of the breast imaging service,* focused on technical performance, quality of practices, and patient-centeredness. Considering the *challenges of technical performance*, the following ten aspects were found: cleaning and artefacts [5, 24, 26], AEC [5, 24], the performance of periodic tests [5, 26], exposure parameters [5, 31], receptor handling [5, 24, 31], screen-film combination [24], viewing conditions [24, 25], breast compression, interprofessional working [24, 25, 27–31, 37, 38], the use of positioning sheets [35], and the implementation of quality control (QC) programs [24, 26, 31] (Table 4).

**Table 4:** Biggest challenges of breast imaging practice according to selected studies

a) Challenges of technical performance
1. cleaning and artefacts: working surfaces, screens, cassettes and processing units
2. AEC: change from manual to AEC, service adjustment to AEC
3. periodic test performance
4. exposure parameters: inappropriate use
5. image receptor: change, damaged or scratched or broken receptors
6. film-screen combination: not spectrally matched or broken
7. viewing conditions
8. breast compression: variations between patients during imaging rounds, between practitioners, imaging sites in association with dose, breast thickness and MGD; use of rigid and flexible compression paddles; different types of practitioner viewpoints and behaviour in using compression force
9. use of positioning sheets
10. implementation of QC programs
b) Challenges of quality of practices
1. positioning
2. image contrast
3. image artefacts
4. variations in image quality in using FS, CR and DR systems
5. use of DRLs: DRLs are defined for standard sized patients which does not always apply to reality
6. implementation of repeat/reject analysis
7. image (FS or DI) processing
8. image labelling and documentation
9. variability in the assessment systems of image quality
c) Challenges of patient centeredness
1. giving seamless and multiprofessional diagnostic services
2. association of heavy workload with deficiencies in patient-centred services: lack of time for the patient
3. promoting breast screening adherence in a radiographer’s work
4. patient-centred viewpoint in the use of compression force

In the selected studies, there were nine types of *challenges associated with the quality of practices* in mammography, comprising the following areas: positioning especially in MLO projection [25, 26, 31, 35]; image contrast [5]; artefacts [25]; the variations in image quality using screen-film, CR and DR systems [33]; the implementation of dose reference levels (DRL) [34]; (a lack of) the implementation of repeat/reject analysis [31]; image processing [26]; image labelling and documentation [25]; and the variability of strategies for image quality evaluation [32] (Table 4).

Challenges of mammography practice associated with the *patients* comprised: (1) the provision of seamless and multiprofessional diagnostic services emphasizing the importance of staff skills and attitude to the quality of breast screening experience [36], (2) a lack of the possibility for the staff to use enough time with the patient due to the heavy workload [31], (3) promoting breast screening adherence in radiographer’s work [33], and (4) the use of compression force [29, 30, 35] (Table 4).

## Discussion

### Challenges of mammography education

According to the European guidelines for quality assurance in breast cancer screening and diagnosis [13, 19], a breast unit must have a core team composed of health professionals of various disciplines who have undergone specialist training in breast cancer beyond that given in their general training. This emphasizes the importance of training in multiprofessional cooperation amongst radiology staff, and the same result was also found in this integrative review [5]. Communication and social skills are also mentioned among the central competences of radiographers in these guidelines [13, 19]. In this integrative review, it was evident that radiographers lacked competence for working in a patient-centred way [25, 36].

According to the selected studies, it is possible to verify the evidence related to the co-existence of both obsolete (SFM) and modern technology (DM) in European countries. This can have an impact on practice and also on radiographer education and training in mammography. The levels of knowledge and the topics to be addressed need to be country specific, promoting harmonization in both areas (clinical practice and training). The training of the students and radiographers should meet the actual needs in their country.

Despite different contexts and equipment, some challenges of education and practice can coexist in several countries, being more related to the lack of motivation and commitment [33]. That was also observed in a previous study related to the continuous professional development of Portuguese radiographers. In that study it was also verified that the area of mammography was less valued in terms of education and training compared to other imaging modalities [39], which may compromise the improvements in this field. Another aspect that has an impact on the quality of mammography services is the heavy workload [31] that may also have an impact on student and educator motivation due to work demands and the lack of time, making education also a challenge under these circumstances.

The analysed studies also showed a concern about basic radiographer training and its sufficiency in order to develop proper technical skills, including accurate positioning, quality control, dosimetry, and teaching skills. The difficulty for our review is that the training differs from one country to another and that the papers do not describe the performance of training in the countries where the studies were performed [5, 24, 25, 31, 32.] Moreover, the educational needs of experienced mammography radiographers must be differentiated from the training needs of the radiographers only starting their work in the breast cancer screening programme. However, the EUREF guidelines and the other European guidelines [31, 32] recommend that radiographers should attend 40 hours of continuous professional development (CPD) training per year, ranging from two to six weeks, depending on the individual performance and experience [31], to improve their basic skills and to stay up to date. Nevertheless, no evidence was found in the selected studies about how many countries follow the European recommendations rigorously. In order to ensure good attendance rates to CPD type of mammography education, it should be made mandatory in all European countries, information about the education opportunities should be properly communicated, and attention should be paid to the pedagogic and technical organization of the education [33].

### Challenges of mammography practice

In this article the challenges of breast imaging practice are divided into three areas: technical performance, quality of practices, and the patient-centred way of working. The challenges related to technical performance and quality of practices like the issues of positioning, breast compression, contrast, noise, artefacts, sharpness, and labelling, have been known for a long time, and identified also in the studies performed outside the European context [26, 32–34, 40–43]. The introduction of digital mammography in the practice brought challenges in breast positioning, mainly due to a bigger breast support according to radiographers [32]. Small breasts and shorter clients are difficult to position without the superimposition of the arm and/or the abdominal wall.

According to Whelehan et al. [44], clients avoid re-attending for screening mammography due to breast pain experienced in the previous exams. During a mammography exam, pain can arise from the application of compression force, being also a challenge in the radiographer practice, as the radiographer rather than the client influences the application of compression force in mammography. The application of compression force and the resultant pain can affect client experience, radiation dose and image quality (noise, sharpness, movement artefacts), and needs to be addressed in radiographer education and training. Mercer et al. [28] also highlighted in their study the considerable variability in breast compression force applied during mammography examination, showing room for improvements in practice and consequently in radiographers’ education. It is crucial to identify strategies allowing the optimisation of breast compression, resulting in pain reduction and improving the image quality.

Technical challenges of the AEC implementation are also common and well known regardless of the cultural context [45]. Other challenges of technical performance that were identified in these European studies involved the performance of periodic quality control tests and the use of exposure parameters and quality control programs in general (Table 4). When the AEC systems are not well calibrated, the selected exposure parameters are affected. Image quality can be insufficient with higher levels of noise, complicating diagnosis, and/or the breast dose can be higher than necessary, affecting the radiation protection of the patient (the dose creep phenomenon). The dose creep can also promote a loss in image quality due to overexposure [31]. However, improvement in quality has been achieved by quantifying quality control results [24].

Some challenges related to the quality of practices, such as DR mammography compared to the indirect CR system from the viewpoints of specificity and sensitivity [43, 46], have been discussed for a long time. The same issues were also observed in our selected studies. Technological changes have an impact on radiographers’ practice in mammography, which must be considered in clinical practice and in education. Some issues regarding the standardization of dose reference levels (DRL) implementation and the challenges caused by the variability in image quality assessment were also emphasized (Table 4). This variability may be due to the fact that although there are uniform guidelines for QA and QC [12, 13, 31], a lack of achievement of the recommended standards in European countries has been identified. There are also variations in the recommendations of the available guidelines, emphasizing the importance of harmonizing the quality keys promoting the achievement of a golden standard for continuous quality improvement. It is also important to update these guidelines periodically, introducing the developments observed in technologies and practices based on evidence [12, 13, 31]. Even when radiographers have the knowledge of main guidelines and the strategies to improve practice, their performance cannot be improved if the healthcare organization does not provide all the resources needed (time, materials, training).

Although communication has been mentioned as one of the central issues in the European guidelines [12, 13, 31], according to the analysed studies, it seems this aspect of mammography practice has not been emphasized as much as the issues of technical quality or the quality of practices. The roles among radiology staff can be very different from one country to another [24]. This is an issue also for radiographers. A deficient image quality that may interfere with the diagnosis should be recognized by the radiographer and corrected, if possible. In addition, offering diagnostic services [36], using time to communicate with patients [31], and promoting breast-screening adherence [33] are topics that should be addressed more. The result is not the desired for the patient when breast cancer is diagnosed, and the experience can be worse if the patient feels emotionally and mentally neglected or disturbed. The quality of screening can be also improved by efficient interprofessional and intraprofessional collaboration and communication [13, 24, 25].

### Implications

- Implementation of the European guidelines for quality assurance in breast cancer screening and diagnosis are varying in European countries. All the European countries should reach the same level of observance.
- Undergraduate radiographer training is not sufficient regarding the quality expectations of the mammography screening programmes.
- CPD education in mammography should have equal demands, and it should be mandatory in all the European countries in order to harmonize the education and clinical practice in mammography.
- Development of mammography training and clinical practice should be considered equal to other imaging examinations performed by radiographers, and resourced to reach the highest quality of education and patient services.
- In addition to technical quality and the quality of practice, the patient-centred viewpoint should also be emphasized both in mammography education as well as in clinical practice.

### Potential biases and limitations of this integrative review

This study was performed because the phenomenon in question is not very well known. The use of a blinded review process and a pre-stated search strategy aimed to control biases in this integrative review. The validity of the results is limited by the variability of the imaging standards in breast screening and mammography in the countries where the selected studies of our review had been performed.

The year limit 2010 proved to be relevant, as anticipated, to getting the studies that describe the most contemporary challenges in mammography education and clinical practice. Due to the limited number of the studies that remained after all the review steps, and due to the specific nature of the topic, a previously modified set of ten criteria was applied that is commonly used to evaluate the quality of reporting studies across different methodologies [22, 23]. A benefit of using this checklist is that the evaluation of the studies can be more easily compared across different types of studies, and this evaluation process can be made visible for readers and evaluators. The used criteria revealed poor rankings in the descriptions of study limitations and generalizability as well as the accounts of addressing potential sources of bias. However, this does not exclude the main limitation of integrative reviews, namely the problem of synthesising findings of studies with disparate methodologies.

## Conclusions

The introduction of harmonized guidelines across Europe may serve as an evidence-based tool to be implemented in practice and education. However, the variability in human and material resources as well as in cultural contexts should be considered to improve education and clinical practice. Training in positioning, QC, image quality evaluation, optimization of breast compression, multiprofessional teamwork, client/radiographer communication, and the patient-centred approach are necessary to meet the challenges of practice identified in the studies analysed in this review.
