# Correction to: European radiographers’ challenges from mammography education and clinical practice—an integrative review

**DOI:** 10.1007/s13244-018-0614-5

**Published:** 2018-04-25

**Authors:** Eija Metsälä, Nicole Richli Meystre, José A Pires Jorge, Anja Henner, Tiina Kukkes, Cláudia Sà dos Reis

**Affiliations:** 1 Metropolia University of Applied Sciences, Helsinki, Finland; 2 Haute Ecole de Santé Vaud, University of Applied Sciences and Arts Western Switzerland, Av. de Beaumont 21, 1011 Lausanne, Switzerland; 3 Oulu University of Applied Sciences, Oulu, Finland; 4 Tartu Health Care College, Tartu, Estonia; 5 Escola Superior de Tecnologia da Saúde de Lisboa, Lisboa, Portugal; 6 Department of Medical Radiation Science, Curtin University, Perth, Australia

**Correction to: Insights Imaging (2017) 8:329–343**

10.1007/s13244-016-0542-1

Affiliation number 2 was rendered incorrectly; the complete and correct affiliation is as follows:

^2^ Haute Ecole de Santé Vaud, University of Applied Sciences and Arts Western Switzerland, Av. de Beaumont 21, 1011 Lausanne, Switzerland.

